# Evaluation of Intraperitoneal [^18^F]-FDOPA Administration for Micro-PET Imaging in Mice and Assessment of the Effect of Subchronic Ketamine Dosing on Dopamine Synthesis Capacity

**DOI:** 10.1155/2022/4419221

**Published:** 2022-10-28

**Authors:** Els F. Halff, Sridhar Natesan, David R. Bonsall, Mattia Veronese, Anna Garcia-Hidalgo, Michelle Kokkinou, Sac-Pham Tang, Laura J. Riggall, Roger N. Gunn, Elaine E. Irvine, Dominic J. Withers, Lisa A. Wells, Oliver D. Howes

**Affiliations:** ^1^Institute of Psychiatry, Psychology and Neuroscience, King's College London, London, UK; ^2^Psychiatric Imaging Group, MRC London Institute of Medical Sciences, London, UK; ^3^Invicro, Burlington Danes, Hammersmith Hospital, London, UK; ^4^Department of Neuroimaging, Institute of Psychiatry, Psychology and Neuroscience, King's College London, London, UK; ^5^Department of Information Engineering, University of Padua, Italy; ^6^Institute of Clinical Sciences, Faculty of Medicine, Imperial College London, London, UK; ^7^Metabolic Signalling Group, MRC London Institute of Medical Sciences, London, UK; ^8^South London and Maudsley NHS Foundation Trust, Camberwell, London, UK; ^9^H. Lundbeck A/S, St Albans AL1 2PS, UK

## Abstract

Positron emission tomography (PET) using the radiotracer [^18^F]-FDOPA provides a tool for studying brain dopamine synthesis capacity in animals and humans. We have previously standardised a micro-PET methodology in mice by intravenously administering [^18^F]-FDOPA via jugular vein cannulation and assessment of striatal dopamine synthesis capacity, indexed as the influx rate constant *K*_*i*_^Mod^ of [^18^F]-FDOPA, using an extended graphical Patlak analysis with the cerebellum as a reference region. This enables a direct comparison between preclinical and clinical output values. However, chronic intravenous catheters are technically difficult to maintain for longitudinal studies. Hence, in this study, intraperitoneal administration of [^18^F]-FDOPA was evaluated as a less-invasive alternative that facilitates longitudinal imaging. Our experiments comprised the following assessments: (i) comparison of [^18^F]-FDOPA uptake between intravenous and intraperitoneal radiotracer administration and optimisation of the time window used for extended Patlak analysis, (ii) comparison of *Ki*^Mod^ in a within-subject design of both administration routes, (iii) test-retest evaluation of *Ki*^Mod^ in a within-subject design of intraperitoneal radiotracer administration, and (iv) validation of *Ki*^Mod^ estimates by comparing the two administration routes in a mouse model of hyperdopaminergia induced by subchronic ketamine. Our results demonstrate that intraperitoneal [^18^F]-FDOPA administration resulted in good brain uptake, with no significant effect of administration route on *Ki*^Mod^ estimates (intraperitoneal: 0.024 ± 0.0047 min^−1^, intravenous: 0.022 ± 0.0041 min^−1^, *p* = 0.42) and similar coefficient of variation (intraperitoneal: 19.6%; intravenous: 18.4%). The technique had a moderate test-retest validity (intraclass correlation coefficient (ICC) = 0.52, *N* = 6) and thus supports longitudinal studies. Following subchronic ketamine administration, elevated *K*_*i*_^Mod^ as compared to control condition was measured with a large effect size for both methods (intraperitoneal: Cohen's *d* = 1.3; intravenous: Cohen's *d* = 0.9), providing further evidence that ketamine has lasting effects on the dopamine system, which could contribute to its therapeutic actions and/or abuse liability.

## 1. Introduction

The radiotracer [^18^F]-fluoro-3,4-dihydroxyphenyl-L-alanine ([^18^F]-FDOPA) has been used to noninvasively study presynaptic dopamine synthesis capacity in the human brain using positron imaging tomography (PET) for nearly four decades [1–4]. [^18^F]-FDOPA PET imaging has been instrumental to demonstrate altered dopamine function due to stress, pharmacological challenges, and the pathology of neurodegenerative disorders and psychiatric illness [3, 5–11]. In schizophrenia, specifically, [^18^F]-FDOPA PET imaging has revealed elevated dopamine synthesis capacity in the striatum of patients as well as those at high risk or in the prodromal phase of schizophrenia [12–17]. Conversely, patients with neurodegenerative disorders like Parkinson's disease show decreased uptake in the same region [18, 19]. Thus, [^18^F]-FDOPA PET imaging is a critical tool in medical research for diagnostics and monitoring disease progression, as well as for elucidating underlying disease pathology and assessing effectiveness of existing and novel pharmacological treatments.

In more recent years, the development of micro-PET technology [20] has enabled the study of small animal models of human disease, allowing for translational investigation of underlying pathology and supporting the development of novel therapies. Mice and rats in particular offer the advantage of being experimentally tractable models, including the generation of phenotypes by pharmacological treatment, genetic models using protein knockout or overexpression, and functional manipulation using optogenetic or chemogenetic regulation [21–23]. Work from our group recently showed that subchronic ketamine treatment in mice resulted in elevated striatal dopamine synthesis capacity, resembling the dopaminergic alteration seen in patients with schizophrenia, and demonstrated that this measure could be reduced using pharmacological treatment [24].

In mice, radiotracers for PET imaging are routinely administered intravenously (i.v.), particularly via the superficial tail vein. However, repeated administration can lead to injury and, due the small bore of this vein, the injectable volume is limited, while partial paravenous injection is a risk that can lead to inadequate radiotracer delivery. Jugular vein cannulation, which enables fast delivery to the brain, is a challenging and invasive procedure in mice which is normally a terminal procedure as recovery after extended anaesthesia is challenging, thus preventing longitudinal assessments. In the case of longitudinal studies, chronic intravenous catheters are technically difficult to maintain and can be stressful to mice. L-3,4-Dihydroxyphenylalanine (L-DOPA) is absorbed in the rat peritoneum with less interindividual variability than oral dosing [25]. However, it is not known if this is the case in mice, where difference in size and metabolism may affect radiotracer uptake in the brain.

In the current study, we investigated intraperitoneal (i.p.) [^18^F]-FDOPA administration to evaluate dopamine synthesis capacity, expressed as rate constant *K*_*i*_^Mod^, in mice in four steps. In experiment 1, we compared [^18^F]-FDOPA uptake between i.v. and i.p. radiotracer administration and optimised the time window used for estimation of *K*_*i*_^Mod^. Experiment 2 compared *K*_*i*_^Mod^ in a within-subject design between i.v. and i.p. routes of administration of the radiotracer. Experiment 3 evaluated the test-retest correlation of *K*_*i*_^Mod^ in a within-subject design of i.p. radiotracer administration. Finally, experiment 4 compared the *K*_*i*_^Mod^ values obtained by the two routes of administration in a mouse model of hyperdopaminergia induced by subchronic ketamine administration. Our results show that i.p. administration of [^18^F]-FDOPA leads to good brain uptake of the radiotracer, moderate test-retest validity, and measurement of dopamine synthesis capacity comparable to the i.v. administration route. Intraperitoneal administration of [^18^F]-FDOPA thus enables longitudinal studies, refined methods to support more humane animal research, and has translational value in aiding development of novel therapies to treat dopamine dysfunction.

## 2. Methods

### 2.1. Subjects

Male wild-type C57BL/6J mice, aged 6-7 weeks old upon arrival, were obtained from Charles River (Kent, UK) and housed in groups of up to 4 under a 12 : 12-hour light dark cycle with food and water provided ad libitum. Animals were habituated for a minimum of 1 week, before being used in procedures at a minimum age of 8 weeks; the overall weight range of animals used in this study was 19-34 g (26.0 ± 2.6 g), and animals were randomly assigned to different experiments and treatment groups. All animal experimental procedures were performed in accordance with the UK Animals (Scientific Procedures) Act 1986 and EU directive 2010/63/EU, and protocols were also approved by Invicro and Imperial College Animal Welfare and Ethical Review Body.

### 2.2. Experimental Design

Experiment 1 compared [^18^F]-FDOPA uptake kinetics between i.v. (*N* = 12) and i.p. (*N* = 13) routes of administration of the radiotracer. Experiments were performed in naïve mice weighing 19-34 g (25.9 ± 3.3 g). Following a bolus injection of 1-19 MBq (8.4 ± 5.6 MBq; 0.040 ± 0.010 GBq/*μ*mol), PET acquisition was carried out for 120 minutes. All mice subjected to an [^18^F]-FDOPA scan with i.p. injection of the radiotracer were allowed to recover at the end of the scan, monitored for 2 days to ensure their welfare, and, following an interval of at least 2 days, reused for subsequent longitudinal scanning in experiment 2 (*N* = 7) or experiment 3 (*N* = 6).

Experiment 2 compared *K*_*i*_^Mod^ following i.p. or i.v. [^18^F]-FDOPA injection using a within-subject design. Following an interval of at least 12 days, a subset (*N* = 7) of the animals recovered from the i.p. injected group in experiment 1 were subjected to a second [^18^F]-FDOPA scan using i.v. administration of the radiotracer. Animals used for this comparison weighed 25-34 g (29.0 ± 2.7 g). Following a bolus injection of 2-13 MBq (7.3 ± 3.5 MBq; 0.039 ± 0.0084 GBq/*μ*mol), PET acquisition was carried out for 120 minutes.

Experiment 3 evaluated the test-retest estimation of *K*_*i*_^Mod^ in a within-subject design of i.p. radiotracer administration. Following an interval of 2 days, a subset (*N* = 6) of mice recovered from the i.p. injected group in experiment 1 were subjected to a second [^18^F]-FDOPA scan using i.p. administration of the radiotracer. Mice used in this comparison weighed 22-25 g (23.7 ± 1.1 g). Following a bolus injection of 5-25 MBq (14.9 ± 5.5 MBq; 0.048 ± 0.0058 GBq/*μ*mol), PET acquisition was carried out for 120 minutes.

Experiment 4 compared the *K*_*i*_^Mod^ values obtained by the two routes of administration in a mouse model of hyperdopaminergia induced by subchronic ketamine administration as described previously [24]. In brief, mice received a daily i.p. injection for 5 consecutive days of 30 mg/kg ketamine (Sigma-Aldrich, K2753) dissolved in saline (KET, *N* = 24) or were left untreated (Ctrl, *N* = 23). Following an interval of 2 days after the last ketamine injection, animals were subjected to a single [^18^F]-FDOPA scan using either i.v. or i.p. administration of the radiotracer. Animals in this study weighed 21-32 g (25.9 ± 2.0 g). Following a bolus injection of 2-23 MBq (9.0 ± 4.8 MBq; 0.043 ± 0.012 GBq/*μ*mol), PET acquisition was carried out for 120 minutes (i.v.) or 140 minutes (i.p.).

### 2.3. PET Acquisition

[^18^F]-FDOPA was synthesised as described previously in the supplementary material published by Jauhar et al. [3]. One hour prior to administration of [^18^F]-FDOPA, mice were anaesthetised with isoflurane to undergo cannulation, and were maintained under isoflurane anaesthesia at 1-2% in 1 L/min oxygen for the duration of the study, with respiration rate and body temperature being monitored continuously throughout (BioVet, m2m Imaging Corp, OH, USA). To allow radiotracer delivery, either the external jugular vein was cannulated 45 minutes prior to scanning or an intraperitoneal cannula was inserted 30 minutes before the scan. In order to prevent peripheral metabolism of the [^18^F]-FDOPA, inhibitors of catechol-O-methyl-transferase (COMT) and aromatic amino acid decarboxylase (AADC), entacapone (40 mg/kg; Sigma-Aldrich, SML0654) and benserazide hydrochloride (10 mg/kg; Sigma-Aldrich, B7283), were given intraperitoneally, respectively, at 45 minutes and 30 minutes prior to administration of [^18^F]-FDOPA [22–24]. To ensure optimal absorption of these inhibitors and the radiotracer and to avoid competition with uptake of other neutral amino acids in diet across the blood-brain barrier (BBB) [24, 26], animals were food-deprived for 45 minutes before being anaesthetised. After administration of the inhibitors, mice underwent a 10 min CT scan in an Inveon PET/CT scanner (Siemens, Surrey, UK) to allow for attenuation correction of the PET signal. A dynamic PET scan was started concomitantly with bolus injection of [^18^F]-FDOPA, and data was collected for up to 140 minutes.

### 2.4. PET Image Processing

Following PET acquisition, data were histogrammed into 43 frames (10 × 3 s, 6 × 5 s, 8 × 30 s, 5 × 60 s, 6 × 300 s, and 8 × 600 s) for PET scans lasting 120 minutes. In the case of scans acquired for 140 minutes (experiment 4, i.p. injected group), two additional frames of 600 s were included. Data were reconstructed using filtered back projection with CT attenuation correction, adjusting for random noise, scatter, and radiotracer decay. Image processing was carried out using the Inveon Research Workspace (Siemens, USA), where the CT image was coregistered to the reconstructed PET image and checked for alignment. For each subject, the percentage-injected dose was normalised for body weight and injected activity to provide standardised uptake values (SUV). Regions of interest (ROI) with predefined shape and size were drawn manually on summation radioactivity images around the left and right striata (square; 0.07 cm^3^ each) and the cerebellum (rectangular; 0.1 cm^3^) to extract time-activity curves (TACs) guided by CT and mouse brain anatomy [27].

### 2.5. Extended Patlak Analysis

Kinetic analyses were performed using an in-house pipeline in MATLAB version R2020a (MathWorks, MA, USA). Extended Patlak graphical analysis was used to determine the rate constant *K*_*i*_^Mod^ (min^−1^), which provides an estimate for the rate of [^18^F]-FDOPA striatal uptake and its subsequent conversion into [^18^F]-fluorodopamine ([^18^F]-dopamine or [^18^F]-FDA). *K*_*i*_^Mod^ is a modified version of the influx rate constant *K*_*i*_^Cer^ (min^−1^) and takes into account an estimation of *k*_loss_ (min^−1^), the rate at which signal is removed from the system due to metabolism of [^18^F]-FDA, as described previously [22, 28–31]. In brief, peripherally administered [^18^F]-FDOPA is transported across the BBB to be taken up into striatal-projecting dopamine neurons and is converted by AADC into [^18^F]-FDA, which then becomes irreversibly trapped in brain tissue [30, 32]. The rate at which [^18^F]-FDOPA is taken up and converted into [^18^F]-FDA is used as an index of presynaptic dopamine synthesis capacity. This rate constant is referred to as *K*_*i*_^Cer^ when the cerebellum is used as a reference region in place of an arterial input function. The ratio between irreversibly trapped radiotracer measured in the striatum versus free radiotracer in the reference cerebellum region takes on a linear relationship when plotted against the ratio of running time integral of cerebellum SUV and cerebellar SUV, a function referred to as stretch time. The slope of this linear plot as derived by graphical Patlak analysis [33] corresponds to the influx rate constant *K*_*i*_^*Cer*^. In humans, [^18^F]-FDA trapping is considered irreversible for up to two hours after [^18^F]-FDOPA administration [32]. Rodents have a much faster metabolic rate, and [^18^F]-FDA is lost through metabolism during typical scan durations [21, 22], resulting in a deviation from the linear relationship between striatal uptake and stretch time. This can be corrected for using an extended Patlak analysis, which provides a modified influx rate constant, *K*_*i*_^Mod^, that estimates the metabolism of [^18^F]-FDA as rate of loss, *k*_loss_ and adjusts for it to restore the linear relationship [24, 29, 30].

### 2.6. Optimal Window for Determining *K*_*i*_^Mod^ and *k*_loss_ Parameters

Accurate estimation of the *K*_*i*_^Mod^ parameter requires the acquisition of sufficient temporal data so as to capture the reversible nature of the kinetics present in the system. Subsequently, *k*_loss_ is determined as the deviation from the linear regression over time. This makes estimation of these two parameters sensitive to the time range chosen to carry out the analysis. To establish the optimal window for determining *K*_*i*_^Mod^ and *k*_loss_ parameters, individual TAC data, acquired following i.p. radiotracer administration in experiment 1 (*N* = 13, acquired up to 120 minutes) and experiment 4 (naïve group only, *N* = 12, acquired up to 140 minutes), was processed through the extended Patlak analysis using varying start (*T*^∗^) and end times (*T*^end^). For each parameter, the standard deviation (S.D.) for *K*_*i*_^Mod^ and *k*_loss_ and the coefficient of variation (%CV) were calculated at the different time points to identify the analysis window that produced the least within-group variability. Using these measures, the optimal time range for analysis of *K*_*i*_^Mod^ and *k*_loss_ following i.v. administration was previously determined to be 15-120 minutes [23]; in the current study, the effect of altering time windows on both parameters was tested for i.p. administration.

### 2.7. Statistical Analysis

Statistical tests were carried out using GraphPad Prism v9 (GraphPad Software, La Jolla, California, USA). Average numerical values in the text are expressed as mean ± S.D.; in graphs, data are shown as mean ± S.D. Data were tested for statistical outliers using ROUT test, *Q* = 0.5%, and tested for normal distribution using the Kolmogorov-Smirnov test to decide on the use of parametric (*t*-test) or nonparametric tests; no statistical outliers were identified, and all data showed a normal distribution. Slopes of SUVR curves were compared using simple linear regression analysis. Statistical tests used are specified in the text and figure legends.

## 3. Results

### 3.1. Experiment 1: [^18^F]-FDOPA Striatal Uptake in the Mouse Brain Is Similar following Either i.p. Or i.v. Radiotracer Injection

#### 3.1.1. Comparison of Radiotracer Uptake

The weight of animals scanned did not significantly differ between groups (i.p., 26.8 ± 3.6 g; i.v., 24.7 ± 2.4 g; *p* = 0.12, *t* = 1.62, unpaired two-tailed *t*-test). However, the dose injected in the i.v. group was lower than that in the i.p. group due to the more restrictive injectable volume using i.v. administration (i.p., 0.45 ± 0.23 MBq/g; i.v., 0.17 ± 0.10 MBq/g; *p* = 0.0018, *t* = 3.574, unpaired two-tailed *t*-test).

**Figure 1:**
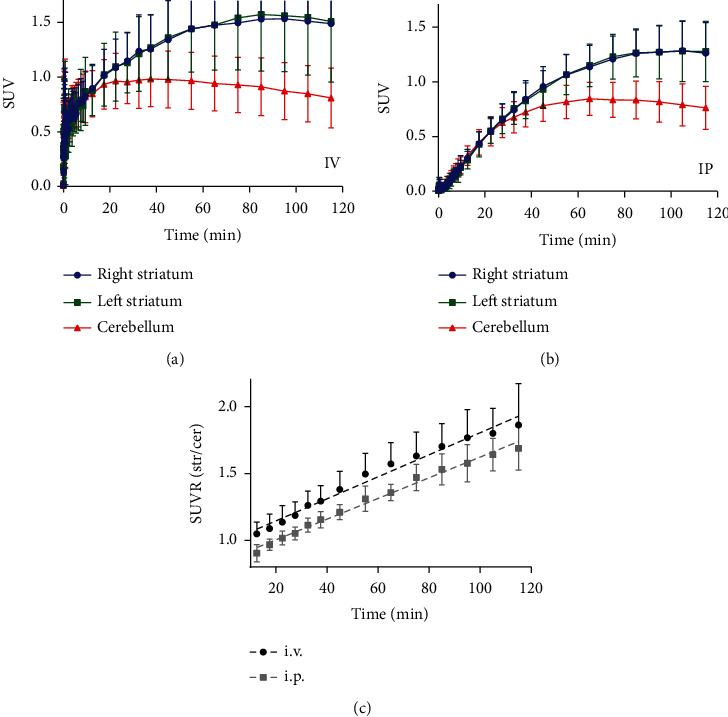
Time-activity curves and SUVR showing the effect of the radiotracer injection method on [^18^F]-FDOPA uptake and loss over time. (a, b) TAC showing SUV of [^18^F]-FDOPA activity over time within the right striatum (blue circles), left striatum (green squares), and cerebellum (red triangles) following intravenous ((a) i.v., *N* = 12) or intraperitoneal ((b) i.p., *N* = 13) administration. Values are mean ±S.D. (c). SUV ratio (SUVR) between the striatum (Str) and cerebellum (Cer) over time following i.v. or i.p. injection. Dashed lines show the best-fit linear regression. Values are mean ± S.D.

The TACs revealed different dynamics of uptake of the [^18^F]-FDOPA radiotracer depending on the injection method (Figures 1(a) and 1(b)). Neither method showed a difference in standard uptake value (SUV) of the radiotracer between the left and right striata (Str) (i.v.: *p* = 0.54, *t* = 0.62; i.p.: *p* = 0.72, *t* = 0.36; paired two-tailed *t*-test). The time to reach the highest level of radiotracer trapping in the striatum (SUV_PEAK_) was slightly delayed following i.p. injection (Figure 1(b)) as compared to i.v. injection (Figure 1(a)), but this did not reach statistical significance (i.v.: 88.8 ± 11.7 min; i.p.: 95.4 ± 14.6 min; *p* = 0.084, *t* = 1.77, unpaired two-tailed *t*-test). Peak striatal SUV levels were reduced following i.p. injection as compared to i.v. injection (SUV_PEAK,IV_, 1.59 ± 0.49; SUV_PEAK,IP_, 1.32 ± 0.26; *p* = 0.017, *t* = 2.47, unpaired two-tailed *t*-test).

In the cerebellum (Cer), where binding of the radiotracer is assumed to be nonspecific and the kinetics are highly reversible, washout started at an earlier time point after radiotracer delivery as compared to the striatum: for both methods, the cerebellar SUV_PEAK_ was reached significantly earlier than the striatal SUV_PEAK_ (i.v._Cer_, 36.2 ± 9.9 min, i.v._Str_, 88.8 ± 11.7 min, *p* < 0.001, *t* = 13.3; i.p._Cer_, 75.8 ± 17.1 min, and i.p._Str_, 95.4 ± 14.6 min, *p* < 0.001, *t* = 3.75; unpaired two-tailed *t*-tests). Furthermore, cerebellar SUV_PEAK_ was reached significantly later following i.p. administration as compared to i.v. administration (i.v._Cer_, 36.2 ± 9.9 min, i.p._Cer_, 75.8 ± 17.1 min, *p* < 0.001, *t* = 7.00, unpaired two-tailed *t*-test). Peak activity levels were significantly lower in the cerebellum than in the striatum for both methods (i.v.: SUV_PEAK, Cer_, 1.01 ± 0.25, SUV_PEAK, Str_, 1.59 ± 0.49, *p* < 0.001, *t* = 3.82; i.p.: SUV_PEAK, Cer_, 0.87 ± 0.18, SUV_PEAK, Str_, 1.32 ± 0.26, *p* < 0.001, *t* = 5.63; unpaired two-tailed *t*-tests). There was no significant difference in cerebellar SUV_PEAK_ values between the two methods (*p* = 0.12, *t* = 1.64, unpaired two-tailed *t*-test).

The ratio between striatal and cerebellar SUV (SUVR) over time is shown for both methods in Figure 1(c). Despite reduced overall SUVR values following i.p. administration, there was no significant difference in slope of the linear fit to the SUVR (calculated for 10-120 minutes) between the two methods (i.v., slope 0.0082 ± 0.00038, *R*^2^ = 0.74; i.p., slope 0.0077 ± 0.00021, *R*^2^ = 0.88; *p* = 0.21, *F* = 1.58).

All mice subjected to an [^18^F]-FDOPA scan with i.p. injection of the radiotracer (*N* = 13) made an unremarkable recovery, enabling their reuse for longitudinal studies.

#### 3.1.2. The Effect of Analysis Window on *K*_*i*_^Mod^ and *k*_loss_ Parameters

**Figure 2:**
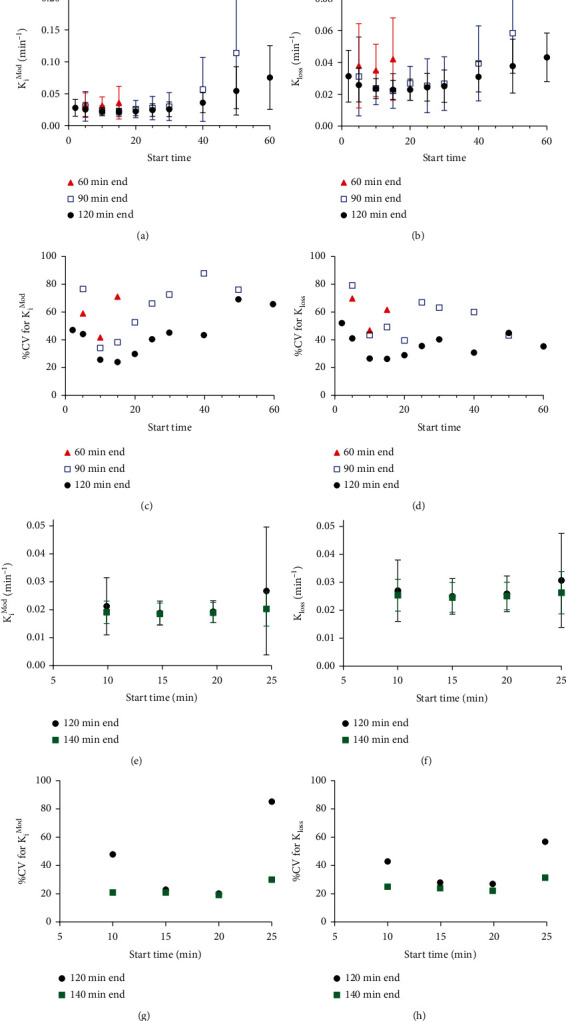
Effect of varying *T*^∗^ and *T*^end^ on *K*_*i*_^Mod^ and *k*_loss_ values and intersample variability following i.p. radiotracer administration. (a, b) Values calculated from TACs following i.p. radiotracer administration (*N* = 13) by the extended Patlak analysis for *K*_*i*_^Mod^ (a) and *k*_loss_ (b) with variable *T*^∗^ (*x*-axis) and *T*^end^ (60 min: red triangles; 90 min: blue open squares; and 120 min: black circles). Values are mean ± S.D. (c, d) %CV for *K*_*i*_^Mod^ (c) and *k*_loss_ (d) with variable *T*^∗^ and *T*^end^. (e–h) As (a–d) but showing the effect of varying *T*^∗^ (*x*-axis) on *K*_*i*_^Mod^ (e) and *k*_loss_ (f) outcome values and S.D. as well as %CV for *K*_*i*_^Mod^ (g) and *k*_loss_ (h) using a *T*^end^ of 120 min (black circles) or 140 min (green squares).

Variation of *T*^∗^ and *T*^end^ in the Patlak analysis of i.p.-administered [^18^F]-FDOPA TACs showed that both the modified influx rate constant *K*_*i*_^Mod^ and the loss rate constant *k*_loss_, as well as the variability within each group, were altered in response to changes in the analysis window (Figures 2(a) and 2(b)). *K*_*i*_^Mod^ and *k*_loss_ could not be determined for analysis window lengths of 30 minutes or less, as in those cases, the estimation of *k*_loss_ would be based on 4 or less points to identify the linear range of the Patlak plot. The percentage coefficient of variation (%CV) for both the *K*_*i*_^Mod^ and *k*_loss_ parameters decreased with increasing *T*^end^, with the least intragroup variability seen with an analysis window of 15-120 minutes (*T*^∗^ and *T*^end^, respectively) (%CV *K*_*i*_^Mod^, 24.4%; %CV *k*_loss_, 26.4%; Figures 2(c) and 2(d)). Consistently, at this time window, the S.D. for both *K*_*i*_^Mod^ and *k*_loss_ is lowest (Figures 2(a) and 2(b)), suggesting the greatest degree of consistency across samples. This is identical to the analysis window optimised for i.v. administration of [^18^F]-FDOPA in previous work [23].

Due to the slower uptake kinetics following i.p. radiotracer administration, resulting in a shorter time window available for the estimation of *k*_loss_, we verified in a separate dataset (*N* = 12 naïve animals) whether extending the scan time and therefore the analysis window used for the estimation of *K*_*i*_^Mod^ and *k*_loss_ by the extended Patlak analysis to 140 minutes could further reduce the within-group variability of *K*_*i*_^Mod^ and *k*_loss_. Assessment of a limited set of *T*^∗^ values showed that at a *T*^end^ of 140 minutes, the within-group variability and %CV are less sensitive to variation in *T*^∗^ than when using a *T*^end^ of 120 minutes, and an analysis window of 20-140 minutes yielded slightly reduced variability compared to 15-120 minutes (%CV *K*_*i*_^Mod^, 19.1%; %CV *k*_loss_, 19.7%; Figures 2(e)–2(h)). Therefore, the analysis window of 20-140 minutes was chosen for analysis of scan data acquired up to 140 minutes following i.p. administration of [^18^F]-FDOPA, whereas an analysis window of 15-120 minutes was selected as most optimal for datasets acquired up to 120 minutes.

### 3.2. Experiment 2: Within-Subject Comparison Shows No Effect of Radiotracer Administration Route on *K*_*i*_^Mod^ Value

Mice underwent two scanning sessions, the first scan following i.p. administration of [^18^F]-FDOPA and the second scan following i.v. administration of the radiotracer, with an interval of at least 12 days between scans. Animals did not significantly differ in weight in consecutive scans (i.p., first scan, 29.1 ± 3.6 g; i.v., second scan, 28.8 ± 1.8 g; *p* = 0.84, *t* = 0.22, paired two-tailed *t*-test) or in injected dose (i.p., first scan, 0.28 + 0.14 MBq/g; i.v., second scan, 0.23 ± 0.10 MBq/g; *p* = 0.48, *t* = 0.75, paired two-tailed *t*-test).

**Figure 3:**
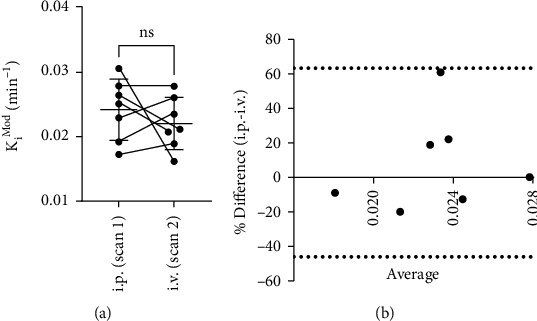
Within-animal comparison of *K*_*i*_^Mod^ outcome values. (a) *K*_*i*_^Mod^ calculated using 15-120 min of the TAC and extended Patlak analysis. Values are mean ± S.D.; *N* = 7; ns: not significant, paired two-tailed *t*-test. (b) Comparison of *K*_*i*_^Mod^ outcome values using a Bland-Altman plot to estimate bias. Dotted lines indicate the 95% limits of agreement.

There was no significant difference between mean *K*_*i*_^Mod^ derived from either method (Figure 3(a); i.p., 0.024 ± 0.0047 min^−1^; i.v., 0.022 ± 0.0041 min^−1^; *p* = 0.42, *t* = 0.87, two-tailed paired *t*-test), and the coefficient of variation was comparable (i.p., 19.6%; i.v., 18.4%). Likewise, there was no difference in *k*_loss_ derived from either method (i.p., 0.024 ± 0.0056 min^−1^; i.v., 0.023 ± 0.0040 min^−1^; *p* = 0.69, *t* = 0.42, paired two-tailed *t*-test; %CV i.p., 23.1%; %CV i.v., 17.0%; data not shown). Comparison of the methods by a Bland-Altman plot (Figure 3(b)) showed a bias of 8.7 ± 28.2% towards i.p. administration; however, this finding did not indicate a consistent bias. However, the order of scans was not counterbalanced, and that is a limitation of this experiment.

### 3.3. Experiment 3: Test-Retest Reliability of Striatal Dopamine Synthesis Capacity to Validate the Use of i.p. Administration of [^18^F]-FDOPA for Longitudinal Studies

Mice underwent two scanning sessions following i.p. administration of [^18^F]-FDOPA, with an interval of 2 days between scans. Between consecutive scans, animals did not significantly differ in weight (first scan, 24.2 ± 0.7 g; second scan, 23.3 ± 1.2 g; *p* = 0.0644, *t* = 2.37, paired two-tailed *t* − test) or in injected dose (first scan, 0.64+ 0.12 MBq/g; second scan, 0.61 + 0.31 MBq/g; *p* = 0.77, *t* = 0.31, paired two-tailed *t*-test).

**Figure 4:**
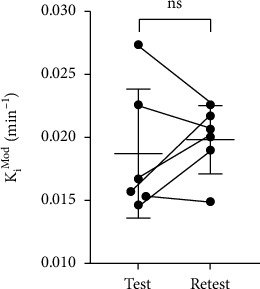
Test-retest reliability of *K*_*i*_^Mod^ outcome values. *K*_*i*_^Mod^ calculated using 15-120 minutes of the TAC and the extended Patlak analysis. Values are mean ± S.D.; *N* = 6; ns: not significant, paired two-tailed *t*-test.

There was no significant difference between mean *K*_*i*_^Mod^ for subsequent scans (Figure 4; test, 0.0187 ± 0.0051 min^−1^, retest, 0.0198 ± 0.0027 min^−1^; *p* = 0.54, *t* = 0.65, paired two-tailed *t*-test), although the coefficient of variation was reduced in the retest scans (test, 27.3%; retest, 13.7%). Likewise, there was no difference in *k*_loss_ derived from either method (test, 0.021 ± 0.0070 min^−1^; retest, 0.016 ± 0.0030 min^−1^; *p* = 0.13, *t* = 1.83, paired two-tailed *t*-test; %CV test, 33.3%; %CV retest, 18.3%; data not shown). Using the two-way mixed model with absolute agreement, the intraclass correlation coefficient (ICC) for *K*_*i*_^Mod^ was 0.515, which indicated a moderate correlation between the two datasets [34]. Likewise, the percentage absolute variability (%VAR) indicated moderate reliability (%VAR = 17.8 ± 10.7%).

### 3.4. Experiment 4: Elevated Dopamine Synthesis Capacity following Subchronic Ketamine Can Be Reproduced Using Either i.p. Or i.v. [^18^F]-FDOPA Administration

*K*_*i*_^Mod^ values in naïve animals (Ctrl) versus animals that received subchronic ketamine administration (KET) were compared following a single scan using either i.p. or i.v. [^18^F]-FDOPA administration. There was no significant difference between treatment groups in animal weight (naïve, 26.3 ± 2.3 g; KET, 25.5 ± 1.8 g; *p* = 0.18, *t* = 1.38, unpaired two-tailed *t*-test) or injected dose (naïve, 0.30 + 0.15 MBq/g; KET, 0.39 + 0.20 MBq/g; *p* = 0.064, *t* = 1.90, unpaired two-tailed *t*-test).

**Figure 5:**
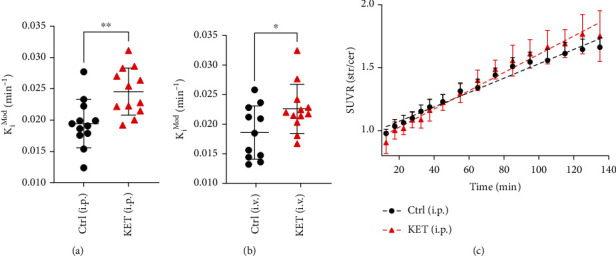
The effect of subchronic ketamine administration on dopamine synthesis capacity measured by two different routes of [^18^F]-FDOPA administration. (a, b) *K*_*i*_^Mod^ calculated using (a) 20-140 minutes of the TAC and extended Patlak analysis following i.p. administration or (b) 15-120 minutes of the TAC following i.v. administration of [^18^F]-FDOPA in naïve (Ctrl) or ketamine-treated (KET) animals. Values are mean ± S.D.; *N* = 12 (Ctrl, i.p.; KET, i.p.; KET, i.v.) or *N* = 11 (Ctrl, i.v.); ^∗^*p* < 0.05, ^∗∗^*p* < 0.01, unpaired two-tailed *t*-tests. (c) SUVR over time in Ctrl or KET animals and following i.p. administration of [^18^F]-FDOPA. Dashed lines show the best-fit linear regression. Values are mean ± S.D.

A significant increase in *K*_*i*_^Mod^ was observed following subchronic ketamine administration as compared to naïve animals, using either i.p. administration (Figure 5(a), Ctrl, 0.019 ± 0.0039 min^−1^, KET, 0.025 ± 0.0038 min^−1^; *p* = 0.0035, *t* = 3.28, unpaired two-tailed *t*-test) or i.v. administration (Figure 5(b), Ctrl, 0.019 ± 0.0045 min^−1^, KET, 0.023 ± 0.0041 min^−1^, *p* = 0.037, *t* = 2.22). For both methods, the difference was observed with large effect size (i.p.: Cohen's *d* = 1.34; i.v., Cohen's *d* = 0.93). The slope of the striatal/cerebellar SUVR following i.p. administration of the radiotracer was significantly increased in ketamine-treated animals as compared to naïve animals (Figure 5(c)) (naïve, slope 0.0057 ± 0.00014, *R*^2^ = 0.90; KET, slope 0.0072 ± 0.00020, *R*^2^ = 0.87; *p* < 0.001, *F* = 35.74). A significant increase in *k*_loss_ was observed following subchronic ketamine administration as compared to naïve animals, using i.v. administration (Ctrl, 0.01696 ± 0.0062 min^−1^, KET, 0.022 ± 0.0039 min^−1^; *p* = 0.0285, *t* = 2.35, unpaired two-tailed *t*-test). However no significant increase in *k*_*loss*_ was observed using i.p administration (Ctrl, 0.02515 ± 0.0052 min^−1^, KET, 0.0269 ± 0.0059 min^−1^; *p* = 0.4491, *t* = 0.77, unpaired two-tailed *t*-test) driven by the control group's relatively higher *k*_loss_ value.

## 4. Discussion

In the current study, we evaluated the use of i.p. administration of [^18^F]-FDOPA in comparison to i.v. administration in mice for the assessment of dopamine synthesis capacity in the striatum. Our data show that i.p. administration is a method that allows excellent striatal radiotracer uptake, with moderate test-retest reliability [34], a similar range of outcome values as compared to i.v. administration, and fast and unremarkable recovery of the animals following imaging, enabling longitudinal studies and within-animal comparison. Importantly, using i.p. [^18^F]-FDOPA administration, we were able to reproduce previously measured enhanced dopamine synthesis in a mouse model of hyperdopaminergia [24]. Our results are in agreement with previous studies in rodents where ^18^F-sodium fluoride (^18^F-NaF) and ^18^F-fludeoxyglucose (^18^F-FDG) radiotracers showed comparable results between i.v. and i.p. administration routes [35, 36].

We applied an extended Patlak analysis for the determination of dopamine synthesis capacity following i.p. [^18^F]-FDOPA administration in mice, showing that both *k*_loss_ and the modified influx rate constant *K*_*i*_^Mod^ are sensitive to the start and end times of the analysis window. The derivation of *K*_*i*_^Mod^ is dependent on the estimation of *k*_loss_, and so using the analysis range that provided the most consistent estimate for *k*_loss_ also yielded the least variable estimates of *K*_*i*_^Mod^. Previously, Holden et al. demonstrated in nonhuman primates that with a longer scan duration (end time of 240 minutes), the *k*_loss_ parameter is insensitive to the start time [29]. With longer scan duration, there are more data available with which to derive an accurate measure of *k*_loss_. Likewise, we found that extending the scan time from 120 to 140 minutes reduced sensitivity of *K*_*i*_^Mod^ and *k*_loss_ for variation in *T*^∗^ (Figure 2). Due to the complexity of maintaining mice under anaesthesia for an extended period, further lengthening of scan time is not desirable, particularly when recovering the animal after imaging. In the present study, we found that the 20-140-minute analysis window provided the lowest within-group variability over the time range tested (%CV *K*_*i*_^Mod^ = 19%, %CV *k*_loss_ = 20%, Figures 2(g) and 2(h)). This is a slight increase as compared to 11% in rats at 180-minute scan duration, where the radiotracer was injected i.v. in the tail [22]. As expected, with shorter scan lengths (up to 120 minutes), the within-group variability in our study increased to over 40% (Figures 2(c) and 2(d)); such high variability would potentially restrict the use of this method to studies with only very large expected between-group effect sizes.

With similar intergroup variability as reported here, future mouse studies using the extended Patlak analysis would require group sizes of at least 10 to detect effect sizes of the same magnitude as the effect we found for ketamine (*d* = 1.4) with >90% power. This can be reduced to *N* = 7 for studies where two within-subject measurements are possible; thus, i.p. administration of [^18^F]-FDOPA could support the reduction of subject numbers in rodent PET imaging studies.

In the present study, we have used a group-housed inbred strain of mouse to reduce within-group individual variability and provide greater resolution of the impact of different analysis start and end times, as well as support reproducibility in the test-retest reliability study and the within-subject comparison of i.p. and i.v. administration of [^18^F]-FDOPA. The within-group variability obtained in our study (19%) is somewhat higher than that seen in human [^18^F]-FDOPA PET imaging studies, where the variability in *K*_*i*_^Cer^ was 10% in the whole striatum, with a range of 6-12% in striatal subregions [37]. Likewise, the test-retest reliability (ICC = 0.52 and %VAR = 18%) in our study is somewhat lower than that reported in humans (ICC = 0.84 and %VAR = 4.5% in human striatum) [37]. These differences probably reflect the far larger size of the striatum in humans, which results in higher resolution images and makes correct placement of the ROI more reliable and reproducible than in mice. Nevertheless, the ICC value in mice indicates moderate reliability [34], supporting the use of this approach.

To our knowledge, we are the first group to apply the extended Patlak analysis to mice scanned with [^18^F]-FDOPA, although others have used the radiotracer in mice to measure overall radiotracer binding at a predefined time point after radiotracer injection [38–40]. Although these studies revealed increased radiotracer binding in the striatum as compared to other brain regions, reliability of this parameter has not been assessed in those studies. A major advantage of kinetic modelling using Patlak analysis is that outcome values correspond directly to those used in human [^18^F]-FDOPA PET imaging [10, 41]. SUVR is an alternative proxy measure that has been used to measure dopamine synthesis capacity in humans [42]. Our data provide a preliminary indication that this output value may also be used as an alternative to *K*_*i*_^Mod^ in the assessment of dopamine function using [^18^F]-FDOPA PET imaging and i.p. radiotracer administration in mice (Figure 5(c)).

The *k*_loss_ values determined by our approach, both the i.v. and i.p. methods, are similar to comparable parameters determined in rats [43] and humans [44, 45] in previous studies, but it has been determined to be an order lower in nonhuman primates [30, 31]. There are no comparator studies in mice with the exception of an earlier publication from our group, and they are most likely related to the species in which they are measured [23]. A methodological consideration for [^18^F]-FDOPA PET scanning in rodents is that administration of COMT and AADC inhibitors is required to prevent peripheral metabolism of the radiotracer and that the between-subject variability in the uptake of these inhibitors underlies the variability in the data. The intraperitoneal injection of these inhibitors just prior to intraperitoneal radiotracer injection may introduce additional variability in radiotracer uptake as compared to intravenous injection. The large surface area of the peritoneal membrane aids rapid absorption; however, the radiotracer is predominantly expected to be transported to systemic circulation through the hepatic portal pathway [46]. Other drug-metabolising enzymes do not play a significant role in the breakdown of DOPA [47], and hence, its brain kinetics are suited our study. Peripheral inhibitors are also used in human [^18^F]-FDOPA imaging, supporting the translational value of this approach in mice [3, 6].

Importantly, using i.p. administration of [^18^F]-FDOPA and in an independent cohort using i.v. administration, we were able to reproduce the previously observed increased dopamine synthesis rate in mice receiving subchronic administration of ketamine [24]. One potential limitation for the study on the effects of ketamine is that the control animals did not receive control injections, in contrast to the ketamine-treated animals. Thus, the design cannot disambiguate whether it is the ketamine or injection that resulted in the group differences. However, the study by Kokkinou et al. [24] used saline injections as the control condition, finding a significant difference with the ketamine group, indicating that the lack of control injections in our control group is unlikely to explain our finding. These results provide further evidence that ketamine has effects on the regulation of dopamine signalling. Furthermore, the high reproducibility of the effect supports the use of subchronic ketamine in mice to create a model of hyperdopaminergia as a proxy of the dopamine signalling abnormality associated with psychotic disorders [15]. Also, the lower SUVR to the cerebellum with i.p. than i.v. might mean that the i.p. approach is less sensitive to detect models of reduced dopamine synthesis capacity, although this effect may be offset by the lower variability with i.p. relative to i.v. Studies using models of reduced dopamine synthesis capacity are needed to evaluate this. It is also interesting to note that the mean *K*_*i*_ values in experiment 2 are similar to those in the ketamine exposed group in experiment 4. This may be an artefact of the smaller sample size in experiment 2 but could also reflect cohort differences, which highlights the value of longitudinal imaging.

## 5. Conclusion

In conclusion, we have demonstrated that the intraperitoneal administration of [^18^F]-FDOPA provides a reliable measure of presynaptic dopamine synthesis capacity with similar values to those obtained with intravenous injection. We also show that ketamine elevates dopamine synthesis capacity, extending previous findings to show this in an independent cohort of animals. Subchronic glutamate receptor antagonism using other pharmacological interventions has also shown to elevate dopamine synthesis capacity and elimination of [^18^F]-FDOPA in rodent and humans thus supporting our findings [44, 48]. Our i.p. administration model finding is consistent with elevated dopamine synthesis capacity while the high control group *k*_loss_ in the i.p. model may have obscured differences in catabolism, and so, further studies are needed. However, it must be mentioned that the ketamine model mimics findings seen with the catabolism and elimination of [18F]-FDOPA in the brain of patients with schizophrenia [45]. Application of this method in mice will allow for longitudinal studies in mouse models that assess the effect of genetic, pharmacological, and environmental changes on the dopamine system. The possibility of within-animal comparison allows for a reduction in animal numbers required, thereby supporting ethical standards in animal research to reduce usage of animals where possible. The method allows for translational measurement of presynaptic dopamine function, thereby providing an important tool in understanding the factors that may ultimately play a role in several neurological and psychiatric disorders as well as supporting the development of novel pharmacological treatments.

## Data Availability

The datasets generated and/or analysed in this study, as well as all codes used in the present study, are available from the corresponding author upon reasonable request.
